# Integrating carers’ experiences and barriers to treatment from ANZAED Credentialed Eating Disorder Clinicians and non-credentialed clinicians in Australia

**DOI:** 10.1186/s40337-026-01811-7

**Published:** 2026-11-03

**Authors:** Madalyn McCormack, Janet Conti, Gabriella Heruc, Siân A. McLean, Katarina Prnjak, Rebecca Barns, Phillipa Hay

**Affiliations:** 1 School of Medicine, Translational Health Research Institute, Western Sydney University, Campbelltown, Australia; 2 School of Psychology, Translational Health Research Institute, Western Sydney University, Campbelltown, Australia; 3 School of Medicine, Eating Disorders and Nutrition Research Group, Translational Health Research Institute, Western Sydney University, Campbelltown, Australia; 4 School of Psychology and Public Health, La Trobe University, Melbourne, Australia; 5 Graduate School of Health, University of Technology Sydney, Ultimo, Australia; 6 Mental Health Services, Campbelltown Hospital, Campbelltown, Australia

**Keywords:** Eating Disorders, Credential, Lived experience, Parents, Carers, Treatment, Mixed methods

## Abstract

**Background:**

Eating disorder (ED) rates are rising in Australia, yet many individuals are not accessing timely or appropriate treatment. A key barrier is the availability of clinicians with the clinical knowledge and training to assess, diagnose, and provide timely, safe, and effective eating disorder treatment. The Australia & New Zealand Academy for Eating Disorders (ANZAED) introduced the ANZAED Eating Disorder Credential (the Credential) to address this challenge and ease the burden on individuals living with an eating disorder, their carers’, and other supporting health professionals. The current study aims to explore parents’ and carers’ experience of eating disorder treatment in Australia provided by Credentialed Eating Disorder Clinicians (credentialed clinicians) and non-credentialed clinicians.

**Methods:**

An exploratory mixed-methods cross-sectional study was conducted involving 51 participants who had cared for a young person, family member, or loved one with an ED who received treatment by a credentialed clinician or a non-credentialed clinician. Participants were recruited via Australian eating disorder services and organisations, with recruitment information distributed through their email and social media platforms. Participants completed a survey with open- and closed-ended questions on their attitudes towards the Credential and their experiences of treatment by a credentialed clinician or non-credentialed clinician. Descriptive statistics and Mann–Whitney U tests were used to examine differences in treatment experiences and perceptions of treatment between participants who had experience with credentialed and non-credentialed clinicians, alongside content analysis of open-ended survey responses.

**Results:**

The Credential was valued among parents and carers, irrespective of whether they had experience with a credentialed clinician. Parents and carers also expressed an overall preference for treatment from a credentialed clinician. However, when considering perceived therapeutic success, credentialing status was considered less important than other factors such as access, collaborative care, therapeutic rapport, and individualised treatment. Further, despite the introduction of the Credential, several barriers to safe and effective ED treatment remain.

**Conclusions:**

The Credential is an important step towards improving the standards of care and treatment experiences for individuals with eating disorders and their families. However, addressing ongoing barriers to access and delivery of care will be critical to realising its full potential.

## Abstract (plain language summary)

Eating disorders are becoming more common in Australia, but many people are not accessing the treatment they need. One reason is that it can be difficult to find clinicians with the appropriate training, knowledge, and experience to provide suitable treatment for eating disorders. The Australia & New Zealand Academy for Eating Disorders (ANZAED) introduced the ANZAED Eating Disorder Credential (the Credential) to address this and ease the burden on individuals living with an eating disorder, their carers, and other supporting health professionals. This study explored how parents and carers experience treatment from clinicians who have the Credential compared with those who do not. Parents and carers valued the Credential, regardless of whether they had experience with a credentialed clinician. They also expressed an overall preference for treatment from a credentialed clinician. However, when it came to the perceptions of factors that matter for successful treatment, parents and carers highlighted the importance of other factors. These included treatment access, collaborative care, therapeutic rapport, and individualised treatment. While the Credential is an important step towards improving the standards of care and treatment experiences for individuals with eating disorders and their families, there are still barriers which need to be addressed so that the Credential can reach its full potential.

## Background

Eating disorder (ED) rates are rising in Australia, with estimates suggesting that 4% of Australians are currently living with an ED [1]. Despite high global burden [2], current research suggests that there is a significant gap between the number of individuals affected by an eating disorder and the number receiving appropriate treatment, with early opportunities to intervene frequently missed. Unfortunately, even when individuals do present for treatment, they often face challenges accessing and receiving appropriate care. More specifically, finding experienced health care professionals with the breadth and depth of clinical knowledge and training to accurately identify all eating disorder symptomology, behaviours, and diagnose and provide empirically supported ED treatment can be challenging [3–5].

Further, in Australia and internationally, many health professionals report receiving suboptimal levels of training on screening, monitoring, and managing eating disorders in their tertiary education and as part of their ongoing professional development [6–8]. Thus, the Australia and New Zealand Academy of Eating Disorders (ANZAED) proposed a credentialing system for Australian ED clinicians to ease the burden on individuals living with an eating disorder, their carers, and other supporting health professionals by allowing them to easily identify mental health professionals and dietitians in Australia who have the minimum standard of knowledge, qualifications, and ongoing supervision and professional development to provide safe and effective treatment [9, 10].

The ANZAED Eating Disorder Credential (the Credential) was developed in collaboration with the National Eating Disorder Collaboration (NEDC) and officially launched in June 2022 [10, 11]. The Credential requires dietitians and mental health professionals to have a minimum of two years of prior clinical experience and have completed introductory and treatment provision training through an NEDC approved course. Clinicians are required to complete a minimum of 15 h of eating disorder relevant ongoing professional development and at least six hours of ED relevant supervision (at least three hours of which must be individual supervision) per year to maintain their Credential [10]. Initially general practitioners (GPs) were only eligible to apply for the Credential as a mental health professional if they were a provider of focused psychological strategies. However, in 2024, ANZAED expanded the Credential creating a new pathway specifically for GPs to recognize the important role they play in the assessment, diagnosis, referral and ongoing medical care for people seeking treatment for EDs [12].

Heruc et al. [11] has described the development and aspirations of the ANZAED Credential. In a series of subsequent papers, the experiences and attitudes of people with lived experience of an ED and health care professionals towards the Credential have been reported [13–17]. However, given the essential yet often under recognized role that carers play in eating disorder treatment [18, 19], it is crucial to also understand their experiences of, and barriers to, eating disorder care in Australia [20–23] as this remains underexplored in the existing literature. Carers, including parents, siblings, and spouses, are often the first to recognise early symptoms of an eating disorder and play a central role in supporting treatment and recovery [21]. However, this role can come at a considerable personal cost. Female caregivers report greater caregiving burden, lower quality of life, and higher levels of depression and anxiety than male caregivers [24] with caregiver depressive symptoms associated with eating disorder severity [25]. Carers commonly experience feelings of guilt, worry, and emotional distress, alongside strain on family and interpersonal relationships [21, 26, 27]. These impacts extend beyond psychological wellbeing, encompassing financial hardship related to treatment costs and reduced workforce participation and adverse effects on physical health [21, 28, 29].

To begin addressing this gap, we conducted an in-depth qualitative exploration of 13 carers’ experiences which found a generally positive regard for the care received from credentialed clinicians where participants expressed a high appreciation for providers who had “detailed insight into the ED” (p.8) and perceived a positive impact on the therapeutic relationship [17]. However, barriers to care were also experienced, such as treatment delays, and a lack of support and understanding in primary and early care professionals.

Building on these findings, the current study aimed to investigate in a larger sample, carers experience of eating disorder treatment in Australia including perceived barriers to care, and compare experiences of those carers whose family member, young person, or loved one received treatment from a credentialed or from a non-credentialed clinician.

## Methods

### Design

This study used a mixed methods design that included: (1) quantitative data collected from closed ended survey questions, and (2) qualitative data collected from participants’ free text responses to open-ended survey questions on perceived barriers to care.

### Participants

Participants were carers (*n* = 51) who had cared for a family member, young person or loved one with an ED who received ED treatment by at least one credentialed clinician (*n* = 26) or a non-credentialed clinician (*n* = 25) either currently or in the past. Table 1 provides details of the characteristics of the participants who took part in this study. Demographic statistics with small sample sizes were not reported for confidentiality reasons. Most carers identified as women. Carers who had experience with a credentialed clinician had a median age in the early 50s and those who had experience with a non-credentialed clinician had a median age in the late 40s. Most participants reported an Oceanic ethnic background, resided in a metropolitan area, were married or living as married and had completed university or a college diploma as their highest level of education. Carers reported a range of employment, with most employed full time or part-time/casually. More than three quarters of participants were caring for someone who was currently experiencing ED symptoms and the most commonly reported ED diagnosis was anorexia nervosa. There were no significant differences in the characteristics of carers who had experience with a credentialed clinician and those who had experience with a non-credentialed clinician (all *p*>.05).

**Table 1:** Characteristics of carers

Characteristic	Treating health professional	
	Credentialed clinician (*n* = 26)	Non-credentialed clinician (*n* = 25)
	Median (IQR)	
Age (years)	52.5 (47–54.3)	47 (44.5–50.5)
	**n (%)**	
**Gender** ^1^		
Women	25 (96.2%)	24 (96%)
**Cultural background** ^1,2^		
Oceanic	13 (50%)	17 (68%)
**Relationship status** ^1^		
Married/Living as married	20 (76.9%)	20 (80%)
**Geographical location** ^1^		
Metropolitan	21(80.8%)	18 (72%)
**Highest level of education** ^1^		
University or college diploma	20 (76.9%)	17 (68%)
**Employment status** ^1^		
Employed full-time	10 (38.5%)	9 (36%)
Employed part-time/casually	10 (38.5%)	10 (40%)
**ED status and diagnosis of the person cared for** ^1,2^		
Currently experiencing symptoms	21 (80.8%)	19 (76%)
Anorexia Nervosa	24 (92.3%)	21(84%)
Other Diagnosis^3^	5 (19.2%)	8 (32%)

^1^*n* < 5 were not included in the table to protect participant confidentiality

^2^ These are not exclusive categories

^3^ ‘Other’ included Bulimia Nervosa, Binge Eating Disorder, Rumination, Avoidant/Restrictive Food Intake Disorder, and Other Specified Feeding or Eating Disorder

### Procedure

The study was approved by the Western Sydney University Human Research Ethics Committee (approval number: H15252). Participants were recruited via email, social media advertisements via Australian eating disorder services and community organisations (namely the SWAN Centre, Eating Disorders Families Australia (EDFA), Butterfly Foundation, Eating Disorders Queensland (EDQ), Eating Disorders Victoria (EDV), Inside Out Institute) and through ANZAED and NEDC membership databases. Recruitment occurred in three waves with two follow-ups occurring following an initial recruitment drive. Participants were provided with an anonymous Qualtrics survey link to participate in a self-report survey about the credential’s impact on ED treatment practices and experiences of care. All participants gave informed consent. Participants were only included in the study if they (a) consented to the study (b) confirmed that they were 18-years-of-age or older and were currently residing in Australia, and (c) they had cared for a family member, young person, or loved one with an ED who received ED treatment by a credentialed clinician or a non-credentialed clinician either currently or in the past. Participation was voluntary and participants were made aware that they could withdraw from the study at any time. At the completion of the survey, participants were thanked for their time and were given the option to provide their contact details to receive a $A20 gift card. They were also invited to express their interest in being contacted by the research team for an interview. Analysis of in-depth data from the interviews is presented in a separate paper [17].

### Measures

The survey comprised a range of open- and closed-ended questions developed by the authors (PH, JC, KP) about their family member, young person, or loved one’s eating disorder and treatment history, experience using the (credentialing) ‘Find a Treatment Provider’ search directory, their attitudes towards the ANZAED Eating Disorder Credential and perceived barriers to seeking and accessing treatment. Questions asked participants about their:

- *Demographics.* Participants were asked about their age, gender, cultural and ethnic background, residential location (including the Australian state or territory they reside in and the geographical setting of their primary residential location), marital status, current employment, and highest level of education.
- *Eating disorder history.* Participants were asked about their family member, young person, or loved one’s eating disorder history including the year their symptoms began, the status of their symptoms (currently experiencing, previously experienced, never experienced), and ED diagnosis.
- *Treatment history.* Participants were asked about the treatment history of the person they care(d) for/support(ed), including the type and duration of treatment received, professionals and/or specialists they received treatment from, geographic setting of care, and wait time to access treatment.
- *Experience using the ‘Find a Treatment Provider’ searchable directory.* Participants were asked to detail their experiences (e.g. access, clinician preferences, general user experience) using the ANZAED Credential’s ‘Find a treatment provider’ search tool.
- *ANZAED credentialed clinician treatment history.* Participants were asked if their family member, young person or loved one had ever received ED treatment from an ANZAED Credentialed Eating Disorder Clinician. Those who answered in the affirmative were asked about their family member, young person or loved one’s treatment history with a credentialed clinician including the type of treatment and professionals and/or specialists seen, geographic setting of care, wait time to access treatment, and length of treatment.
- *Attitudes towards the Credential.* All participants, regardless of whether they had experienced treatment with a credentialed clinician or not, were asked about their attitudes towards the Credential. Participants were asked to rate their agreement with statements regarding their attitudes towards credentialed clinicians using a 6-point sliding scale from 0 (strongly disagree) to 5 (strongly agree). An example item is “I place greater trust in the advice of a Credentialed Eating Disorder Clinician than a non-credentialed clinician.” Responses to items were analysed separately.
- *Perceived barriers to seeking and accessing treatment.* Participants were asked about the perceived barriers that affected their access to or use of outpatient or inpatient ED treatment using a 5-point scale ranging from 1 (no impact at all) to 5 (extremely impactful). Participants were also provided with the option to provide a free text response if there were other barriers not listed.

### Analysis

Data were collected and analysed in Microsoft Excel (version 2308) and Statistical Package for the Social-Sciences software (SPSS; version 29). Data were cleaned and inspected for the normality of distributions. The skewness and kurtosis results and a review of histograms found that scores on sliding scale questions were non-normally distributed, requiring non-parametric tests for analyses. Descriptive statistics using frequencies and proportions were used to explore treatment experiences and perceptions of treatment by a credentialed or non-credentialed clinician. Mann Whitney U-tests were used to examine between groups differences in perceptions and experiences of treatment.

Participants were excluded from the analyses if they did not answer any questions that addressed the research question (i.e., only completed demographic data) (*n* = 1) or if they were unsure about whether they had seen a credentialed clinician (*n* = 16). Participants who completed at least one research question, even if they did not complete the entire survey, were included in the study. However, their responses were only included in the analysis for the specific questions they completed. As a result, the sample size varied across analyses.

## Results

### Attitudes towards the Credential

When comparing attitudes toward the Credential, there were no significant differences between participants whose family member, young person, or loved one had seen a credentialed clinician and those who had seen a non-credentialed clinician (see Table 2). Across both groups, the vast majority of participants preferred treatment from a credentialed clinician, reported greater trust in the advice of a credentialed clinician, valued the existence of the Credential, and agreed that the Credential will make specialised care more accessible and improve health outcomes for people with eating disorders. As participants were asked about their attitudes towards the Credential rather than their direct experiences with credentialed and non-credentialed clinicians, it is unclear whether these attitudes are informed by differences in their experiences of care or reflect general perceptions about the Credential. When examining whether the perceived success of therapy was influenced by seeing a credentialed versus a non-credentialed clinician, the distribution of responses varied across groups, indicating mixed views on whether credentialing affects perceived therapy success. Specifically, among participants whose family member, young person, or loved one had seen a credentialed clinician, almost half (47.8%) believed that the success of therapy is influenced by whether treatment is provided by a credentialed or non-credentialed clinician. This view was less common (29.2%) among participants whose family member, young person, or loved one received treatment from a non-credentialed clinician. However, this did not reach statistical significance. 

**Table 2:** Participant responses to questions about their attitudes towards the Credential

Survey questions and response options	Response distribution (%) of participants who have seen a credentialed clinician (*n* = 23)	Response distribution (%) of participants who have seen a non- credentialed clinician (*n* = 24)	Mann- Whitney U Z (*p*)
In terms of the success of therapy, it makes no difference whether they receive treatment from a Credentialed or non-Credentialed Clinician.			-0.73 (0.468)
*Strongly disagree*	21.7	12.5	
*Disagree*	26.1	16.7	
*Somewhat disagree*	8.7	12.5	
*Somewhat agree*	4.3	20.8	
*Agree*	26.1	25.0	
*Strongly agree*	13.0	12.5	
I would prefer for them to receive treatment from a Credentialed Clinician over a non-Credentialed Clinician			-0.24 (0.813)
*Strongly disagree*	4.3	0	
*Disagree*	4.3	4.2	
*Somewhat disagree*	4.3	12.5	
*Somewhat agree*	17.4	12.5	
*Agree*	21.7	29.2	
*Strongly agree*	47.8	41.7	
I place greater trust in the advice of a Credentialed Eating Disorder Clinician than a non-Credentialed clinician			-0.18 (0.859)
*Strongly disagree*	0	0	
*Disagree*	4.3	4.2	
*Somewhat disagree*	17.4	16.7	
*Somewhat agree*	13.0	12.5	
*Agree*	26.1	33.3	
*Strongly agree*	39.1	33.3	
I value the existence of a Credential that recognises the expertise and training of clinicians and requires a commitment to engagement in continual professional development and supervision specific to the treatment of eating disorders.			-0.06 (0.952)
*Strongly disagree*	0	0	
*Disagree*	0	8.3	
*Somewhat disagree*	0	4.2	
*Somewhat agree*	8.7	4.2	
*Agree*	34.8	20.8	
*Strongly agree*	56.5	62.5	
The Credential will make it easier for people with an eating disorder to readily access specialised care.			-0.48 (0.629)
*Strongly disagree*	8.7	0	
*Disagree*	0	8.3	
*Somewhat disagree*	4.3	12.5	
*Somewhat agree*	26.1	12.5	
*Agree*	21.7	16.7	
*Strongly agree*	39.1	50.0	
The Credential will improve the health outcomes of eating disorder patients.			-0.54 (0.590)
*Strongly disagree*	0	0	
*Disagree*	13.0	4.2	
*Somewhat disagree*	17.4	16.7	
*Somewhat agree*	13.0	12.5	
*Agree*	26.1	37.5	
*Strongly agree*	30.4	29.2	

### Treatment history and experiences

Parent and carer respondents were asked about their family member, young person, or loved one’s treatment with a credentialed (*n* = 26) or non-credentialed clinician (*n* = 25). Among those who had experience with at least one credentialed clinician, either currently (65%) or in the past (35%), the most commonly reported pathway to finding a credentialed clinician was through a GP or other health professional (50%), referrals from family, friends or another carer (15%), independent advertising (10%), and other or unknown referrals (25%). From the time participants first sought treatment with a credentialed clinician for their family member, young person, or loved one, they reported a median wait time of 1.8 months (IQR = 1–3) and a median treatment duration of 24 months (IQR = 12–30). Treatment was accessed in person by the vast majority of participants (95%) and/or via Telehealth (70%). Participants could select multiple credentialed professionals involved in treatment; consequently, percentages are not mutually exclusive. The credentialed professionals or specialists most commonly involved in providing treatment were dietitians (42.1%), psychologists (52.6%), and psychiatrists (57.9%).

For parents and carers who had experience with a non-credentialed clinician, most reported that their family member, young person, or loved one received treatment from a dietitian (76%), psychologist (80%), or psychiatrist (76%), either currently (52%) or in the past (48%). The median wait time to access treatment was 1 month (IQR = 1–3) and most treatment occurred in a metropolitan setting (84%). The most commonly experienced treatment modalities were individual therapy (68.2%), Family Based Treatment (54.5%), inpatient therapy (59.1%), nutritional counselling and physical health counselling (45.5%), and medical or psychiatric treatment (63.6%).

Participants’ ratings of barriers affecting their family member, young person, or loved one’s access to or use of outpatient or inpatient eating disorder treatment are shown in Table 3. There were no significant differences in overall ratings of barriers between participants who had seen a credentialed clinician and those who had seen a non-credentialed clinician. However, inspection of the distributions between response categories indicated a statistical trend (*p*<.1) for participants with experience of a credentialed clinician to rate social/work barriers as extremely impactful more so than those with experience of a non-credentialed clinician.

Participants were asked to specify if there were other barriers to treatment. Parents and carers, irrespective of whether they had experience with a credentialed or non-credentialed clinician highlighted the lack of autonomy or control over the treatment available to their family member, young person, or loved one – *“age of daughter and having no control on decisions”; “being zoned for a public hospital that we do not think is good but having no choice but having to go there”.* They also indicated that their family member, young person, or loved one’s readiness to engage in treatment was a barrier to accessing support as well as the impact on their own personal or familial circumstances (e.g. finances, marriage, family, lack of support networks). Participants described a *“fractured public health service*,*”* noting a lack of public treatment options to be referred to. They also highlighted that access needed to extend beyond simply finding treatment, to *“accessing the right professionals”* with the appropriate knowledge and expertise to provide safe and effective care. Further, treatment was perceived to need to be flexible and tailored to the individual and their family’s unique situation and preferences.

This extended to preferencing care that considered the whole child, including their broader circumstances and comorbid conditions. One parent noted that no appropriate support was available for their adolescent, given their family’s particular structure. Another parent described their treatment team’s refusal to consider that their child may have autism:

*“The inability of the inpatient treating team to consider autism as a possibility severely hindered my daughter’s ability to be supported appropriately. The treating team caused more harm than good.”*

Some parents and carers with experience of a non-credentialed clinician also reported a lack of collaboration, both within the treatment team and between the treatment team and the family, as a factor that made accessing and use of eating disorder treatment more difficult – *“Doctors not communicating all options effectively and not communicating with nurses and each other effectively. Doctors not listening to the parents”.*

**Table 3:** Participant responses to questions about the barriers to treatment

Survey questions and response options	Response distribution (%) of participants who have seen a credentialed clinician (*n* = 22)	Response distribution (%) of participants who have seen a non- credentialed clinician (*n* = 23)	Mann- Whitney U Z (*p*)
Please rate how each of the following barriers affected your loved one’s access or use of outpatient or inpatient eating disorder treatment:			
Cost			-1.13 (0.260)
*No impact at all*	13.6	30.4	
*A little impact*	18.2	13.0	
*Moderately impactful*	13.6	17.4	
*A lot of impact*	13.6	8.7	
*Extremely impactful*	40.9	30.4	
Stigma associated with eating disorder			-0.90 (0.367)
*No impact at all*	22.7	26.1	
*A little impact*	36.4	17.4	
*Moderately impactful*	13.6	13.0	
*A lot of impact*	9.1	4.3	
*Extremely impactful*	18.2	39.1	
Inaccessible treatment			-1.20 (0.229)
*No impact at all*	4.5	17.4	
*A little impact*	4.5	0	
*Moderately impactful*	18.2	26.1	
*A lot of impact*	4.5	4.3	
*Extremely impactful*	68.2	52.2	
Social/work barriers			-1.68 (0.092)
*No impact at all*	9.1	26.1	
*A little impact*	13.6	17.4	
*Moderately impactful*	13.6	21.7	
*A lot of impact*	18.2	4.3	
*Extremely impactful*	45.5	30.4	
GP/health professional ED knowledge			-0.63 (0.530)
*No impact at all*	18.2	21.7	
*A little impact*	13.6	21.7	
*Moderately impactful*	22.7	26.1	
*A lot of impact*	22.7	4.3	
*Extremely impactful*	22.7	26.1	
Personal ED knowledge			-0.48 (0.628)
*No impact at all*	9.1	17.4	
*A little impact*	18.2	8.7	
*Moderately impactful*	36.4	30.4	
*A lot of impact*	27.3	13.0	
*Extremely impactful*	9.1	30.4	

## Discussion

This study explored parent and carers’ ED treatment experiences with credentialed and non-credentialed clinicians in Australia. The findings highlight both the potential value of the Credential and the broader systemic challenges that continue to impact access to timely and effective ED care.

The Credential was generally valued and held in favourable regard, even among those parents and carers who had not directly experienced credentialed clinical care. Further, the vast majority of participants indicated that they would prefer treatment from a credentialed clinician. However, decisions about whether to engage a credentialed or non-credentialed clinician may be constrained by factors such as clinician availability and wait times for treatment. Increasing the availability of credentialed clinicians may enable parents and carers to consider the credential status of clinicians as part of their decision-making when accessing treatment.

Those who experienced treatment with a credentialed clinician perceived treatment success more likely with a credentialed compared to a non-credentialed clinician. The opposite was reported by those who experienced treatment with a non-credentialed clinician. However, these findings did not reach statistical significance and should be understood in the context that there are many other perceived factors contributing to treatment success. In our previous research [17], it was suggested that parents and carers place greater emphasis on factors other than clinician credentialing, including access to treatment, collaborative care, therapeutic rapport, and individualised treatment. The perceived success of therapy may also differ between consumers, carers, and health professionals. For carers in particular, the success of therapy may look beyond a reduction of symptoms and weight restoration to include the ability to reclaim their lives outside of the ED, offering hope, cognitive flexibility, and emotional wellbeing [20, 30].

Consistent with previous literature [4, 17, 31–33], parents and carers reported several barriers to safe and effective ED treatment including access, social and/or work barriers, cost of treatment, lack of autonomy in treatment decision making, readiness for change, and personal or familial circumstances. These perceptions did not differ between respondents with experience of a credentialed or non-credentialed clinician, with the exception that carers who experienced treatment from a credentialed clinician rated social/work as more likely barriers. The majority of parents and/or carers were caring for someone with anorexia nervosa, for which Family Based Treatment (FBT) is a first line intervention [34]. FBT places substantial demands on caregivers that may have implications on their social [35, 36] and occupational functioning [28]. For all participants, parents and carers reported health professionals’ attitudes and a lack of collaboration that made accessing and engaging in eating disorder treatment more difficult. This supports Sinclair et al. [17] whereby carers commonly reported difficulties accessing early intervention due to unhelpful interactions, clinicians’ failure to detect initial ED symptoms, and feeling their concerns were dismissed or unacknowledged.

The present study found that referrals to credentialed clinicians most commonly came from GPs or other health professionals, highlighting the critical role that these clinicians play in the early identification and intervention of eating disorders. Given that many health professionals in Australia report receiving suboptimal levels of training in eating disorders [6–8], these findings suggest that increased awareness of the Credential may facilitate referral to appropriately trained and experienced clinicians. Moreover, improved education around the knowledge and skills required to recognise eating disorder symptoms, conduct assessments, and provide timely referrals could also bridge the treatment delay commonly observed among those with eating disorders [32] and ensure families benefit from credentialed clinicians.

This study has several limitations to consider. The sample of carers was predominately women who resided in metropolitan areas and were married and/or living as married, and most participants reported experiences with anorexia nervosa. There was limited representation of individuals from culturally and linguistically diverse backgrounds, and a lack of representation of Aboriginal and Torres Strait Islander peoples. It is also important to consider selection bias, as carers with strong opinions of the Credential may have been more likely to participate in the research. Additionally, because our survey did not capture whether participants had received treatment exclusively from credentialed clinicians, non-credentialed clinicians, or both, participants perceived barriers to treatment with a credentialed or non-credentialed clinician should be interpreted cautiously. Some participants may have considered multiple treatment experiences and others may not have had experiences with either type of clinician, limiting direct comparison. Given that the Credential is still in its infancy, it is also possible that perceptions of the Credential will change over time. Finally, small participant numbers may have led to Type 2 error, in particular where there was a nonsignificant finding of an observation of greater perception of therapy success with a credentialed clinician by those who had received care from a credentialed clinician.

Future research is recommended to continue to explore the impact of the Credential on eating disorder treatment experiences and outcomes. In particular, it would be important to explore relationships between specific caregiving roles (e.g., parent, sibling, partner, or friend) and perceptions of treatment, including views of the Credential. Also, carers’ knowledge or understanding of the training and competency requirements associated with the Credential would be relevant to explore as it may influence perceptions of clinician expertise and treatment preferences. Finally, research should also investigate the extent to which variations in referral pathways, treatment experiences, or clinician credentialing status may impact differences in carers’ perceptions of the Credential.

In conclusion, this study found that parents and carers value the Credential and prefer treatment from credentialed clinicians. However, several barriers that affect treatment seeking and uptake generally, also affect eating disorder treatment with credentialed clinicians. Addressing these barriers and enhancing public awareness of the Credential is important if the Credential is to realise its full potential. While the Credential will not overcome all challenges that arise within eating disorder treatment, it is an important step towards improving standards of care and improving treatment experiences for individuals with eating disorders and their families.

### About this supplement

About this supplement: This article has been published as part of Journal of Eating Disorders Volume 13 Supplement 1, 2025: The Australia & New Zealand Academy for Eating Disorders. The full contents of the supplement are available online at:https://jeatdisord.biomedcentral.com/articles/supplements/volume-13-supplement-1

## Data Availability

In line with the ethics approvals for this study, the datasets used and/or analysed during the current study are not publicly available as they contain information that could compromise the privacy and confidentiality of research participants.

## Abbreviations

ANZAED: Australian and New Zealand Academy of Eating Disorders
ED: Eating Disorder
EDFA: Eating Disorders Families Australia
EDQ: Eating Disorders Queensland
EDV: Eating Disorders Victoria
FBT: Family Based Treatment
GP: General Practitioner
NEDC: National Eating Disorder Collaboration

## Abbreviations

ANZAED: Australian and New Zealand Academy of Eating Disorders
ED: Eating Disorder
EDFA: Eating Disorders Families Australia
EDQ: Eating Disorders Queensland
EDV: Eating Disorders Victoria
FBT: Family Based Treatment
GP: General Practitioner
NEDC: National Eating Disorder Collaboration
